# Novel Solutions to Student Problems: A Phenomenological Exploration of a Single Session Approach to Art Therapy With Creative Arts University Students

**DOI:** 10.3389/fpsyg.2020.600214

**Published:** 2021-01-18

**Authors:** Elizabeth Wilson

**Affiliations:** Creative Arts and Music Therapy Research Unit, Faculty of Fine Arts and Music, University of Melbourne, Melbourne, VIC, Australia

**Keywords:** creative arts, art therapy, single-session therapy, SSAT, youth mental health, student wellbeing

## Abstract

Within the Australian university context, research has uncovered increasing levels of psychological distress, in the form of stress, anxiety and depression. Higher rates of psychological distress have been reported in undergraduate students specifically enrolled in creative arts programs. Despite these increasing levels of psychological distress, university students are reluctant to engage with mental health and wellbeing supports. To explore ways to meet the mental health and wellbeing needs of creative arts university students, the Creative Arts and Music Therapy Research Unit at The University of Melbourne commenced a project exploring the benefits and pitfalls of a brief creative arts therapies approach for students attending a campus based wellbeing clinic. This exploratory research study formed the art therapy component of this much broader research endeavor. Creative arts students in this research study were invited to participate in a single session art therapy encounter that involved the visual exploration of the miracle question, asking students to visually depict “what the problem looks like and how it will look when the problem is resolved or you feel like you can cope with it better?” The descriptive findings of this exploratory research study revealed how the combination of art therapy used within a single session framework was able to afford students a novel means to externalize problems, leading students to forming a less internalized view of the self.

## Introduction

### Increasing Mental Health and Wellbeing Needs of University Students

Research mapping the counseling trends within Australian universities suggests growing numbers of students are presenting with psychological distress in the form of stress, anxiety and depression (Stallman, 2012; O’Keeffe, 2013; Baik et al., 2018). Despite increasing levels of psychological distress, students avoid seeking help in the form of mental health and wellbeing support (Burns and Birrell, 2014).

Within the youth mental health literature, research has demonstrated that when young people decide to engage with mental health services, a “mismatch” often unfolds between youth preferences for brief and collaborative styles of engagement (Gee et al., 2015), and the medical model approaches used within the youth mental health sector (Burns and Birrell, 2014). Mental health literature exploring the therapeutic preferences of young people suggests that first therapy encounters may not be the most appropriate time to undertake medical model intake and assessment protocols (Gulliver et al., 2010; Watsford and Rickwood, 2015). The use of such problem focused assessments may exacerbate young people’s existing negative views of the self, leading to feelings of having been negatively judged and subsequent disengagement from support (Sukhera et al., 2017).

Engaging young people as “active agents” in first therapy encounters is said to require a paradigm shift on behalf of youth mental health clinicians away from assuming the role of “expert” to becoming a “partner” (Talmon, 1990). The single session approach to therapy is being trialed in youth mental health contexts here in Australia, not only as a way to more efficiently meet the needs of young people, but also to more adequately meet young peoples’ preferences for brief and more collaborative styles of mental health support (Perkins, 2006).

### Single Session Therapy: Philosophies and Techniques

The term single session therapy (SST) refers to a single session intervention that has been either previously scheduled or is provided in a drop-in counseling service (Hymmen and Stalker, 2013). As a therapeutic framework, SST offers a lens of practice that stands in contrast to traditional psychotherapies that use first therapy encounters for the purpose of expert assessment of the client’s presenting problems. Psychologist and founder of the SST approach, Moshe Talmon, argues that therapists are often trained in the medical model of practice that encourages the use of the first session to focus on gathering past historical content such as diagnostic history, as opposed to actively attending to important relational processes in the “here and now” (Talmon, 1990).

### SST Is a Flexible Model

One of the key benefits of SST is that it can offer therapists from all therapeutic backgrounds a flexible framework for the integration of a diverse range of therapeutic techniques (Rosenbaum, 1994). The late Art Therapist Shirley Riley (1999) found that combining art therapy within a brief therapeutic framework offered young people an active means to problem solve, using the artwork as a container. Inspired by Riley’s brief integrated approach to youth therapy, this research study explored the integration of art therapy used within a single session framework, for use with creative arts university students.

### Research Gaps

Existing research in the field of SST has predominantly focused on client outcomes such as “client satisfaction” or “symptom improvement,” rather than understanding from the client’s perspective which aspects of the therapy clients describe as being important (Campbell, 2012). Existing SST research is also yet to explore the integration of creative arts therapies within this model, including what the benefits and pitfalls of this combined approach might look like from the client perspective. This research sought to gain an “insider’s view” regarding how creative arts students described their single session art therapy (SSAT) encounters. Gathering such descriptions may help support the reconfiguration of existing student mental health services, so as to more collaboratively engage students with campus based mental health and wellbeing supports. What follows is a condensed description of the methods used by the therapist/researcher to conduct this study.

## Methods

### Research Location

**FIGURE 1:**
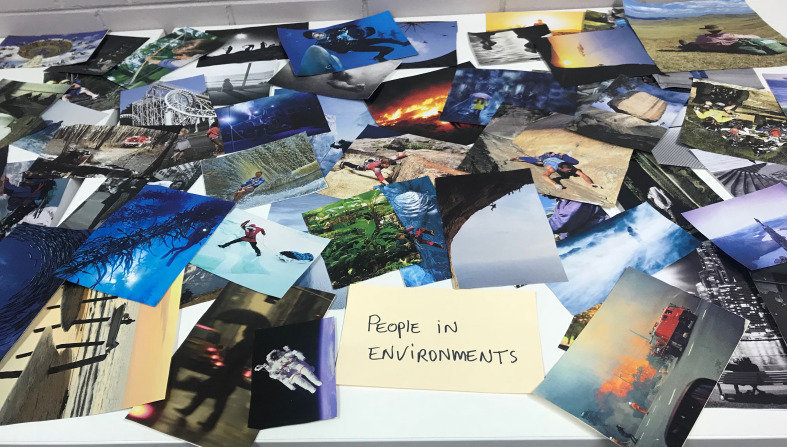
The pre-cut image buffet for single-session art therapy.

The location of this study was in a room called the CAT Lab (Creative Arts Therapies Lab) at a university campus. This room was retrofitted for the purposes of conducting creative arts therapies research and thus provided students with a suitably confidential space. The CAT lab had two therapy chairs and set of long tables on which to place collage art materials. The researcher used these long tables to display a vast array of pre-cut images to use in the Miracle Question Collage Activity (Figure 1).

### Participants

A convenience sample of six creative arts university students was recruited from a larger research study exploring the benefits and pitfalls of a creative arts therapies wellbeing clinic for students. A small sample size was chosen for the purposes of this interpretive research design. All six students identified as being female and were aged between 20 and 30 years. Five of the women were from an English-speaking Caucasian background, with one student of Asian descent having English as a second language. It has previously been mentioned that creative arts students have reported higher levels of psychological distress than any other faculty, and thus this population of students were targeted for inclusion in this study.

### Single Session Art Therapy (SSAT) Procedures

#### Pre-session Phone Call

Prior to meeting with students in person, students received a phone call to determine the key problem the students wished to process during the SSAT encounter. Students were then invited to attend an in person, 90-min session at the creative arts therapies lab. These sessions involved the following activities:

#### Activity 1: Create a Collage Response to the “Miracle Question” (30 min)

The first activity involved students selecting images and or drawing to create a visual response to the following question:

“Think of the specific problems that you are facing that you feel comfortable exploring today. Holding that problem in your mind, select images from the buffet to depict what living with the problem looks like, and how it might look when the problem no longer exists or you feel like you can cope with it.” (Riley, 1999).

#### Activity 2: Verbal Intuiting With the Collage Image (30 min)

Students were invited in a conversation about their completed collage image using the open-ended question “what do you see?” (Betensky, 1995). Further open ended questions that were used during this conversational process were informed by the brief art therapy work of Matto et al. (2003) and included such questions as:

• “What image stands out most for you?”
• “What part of the image is most inviting?”
• “What part is most challenging?”
• “What would you change about the image?”
• “Who would you share this image with?”
• “Who would you not share this image with?”
• “Do you experience any physical sensations when you look at your image?”

#### Activity 3: Open-Ended Interview Evaluation (30 min)

The aim of this evaluation was to have participants describe their immediate therapy experiences to gather rich descriptions of the therapy. The following questions were used as interview prompts:

• “How was this experience for you?”
• “What if anything was beneficial?”
• “What if anything was difficult?”
• “What was I doing as the therapist/researcher that may have been helpful or unhelpful?”

#### Activity 4: One Month Follow Up Interview (60 min)

The follow up interview served two core research purposes. The first was to understand any lasting impressions of their SSAT encounters. Participants were also invited to look at their collage images again for a second viewing, to see if anything had shifted in their interpretation since the first encounter. To gather this information the following questions were asked of students:

• “When you look at your artwork now, what do you see?”
• “Reflecting back on the encounter experience, does anything stand out for you?”

### Research Design

An interpretive phenomenological analysis (IPA) was the chosen design for use in this research project, as it is considered a valuable tool for psychological research that aims to understand the core experiences of clients (Smith and Osborn, 2003; Creswell and Poth, 2018). IPA was therefore selected as an appropriate design to use in this study as the aim was to form an in-depth understanding of students’ lived experiences of their SSAT encounter.

### Data Analysis

The researcher produced transcripts from listening and watching audio-visual recordings of each SSAT encounter with the participants. In order to obtain a full understanding of students’ experiences, the researcher then engaged in a process of crafting six encounter stories as a means of data dwelling. Descriptive themes from these stories and transcripts were then formulated.

## Results and Discussion

### Research Theme: SSAT as a “Novel Experience”

The creative arts research students described the SSAT encounter experience as “novel.” Novelty is described in the literature on creative thinking as encompassing any idea, process or product deemed by a perceiver as offering a feeling of “departure from the familiar” (Gillam, 2018). Within the field of brief psychotherapy, novelty is considered to help “disorient clients in positive ways” through the therapist’s strategic use of techniques that help stimulate a sense of play, humor and imagination in the therapy space (Steenbarger, 2006). The use of novelty in therapy is said to be an important ingredient of change, as it can help stimulate mental flexibility, needed for clients to be open to moments of perspective change (Steenbarger, 2006).

Helping to instill novelty within SSAT encounters with students in this research study was a complex interplay of factors. Important to understand in the context of this study, was that all six students were from a creative arts academic background. Hence, students arrived to the SSAT encounter space with a propensity for seeing and appreciating novel strategies used by the therapist/researcher to stimulate imaginative exploration.

The students in this research study found that the opportunity to explore the miracle question using collage imagery was a novel experience. These students were mostly from the dramatic arts, and were accustomed to constructing narratives using words as opposed to using found images. The experience of novelty driven by the use of images is best captured in the following descriptions of the SSAT encounter:

• “Collage is my least practiced art form…so it was awesome to exercise my mind creatively…doing something I’m not familiar with necessarily” – Joan
• “I appreciated this experience because it was a different way to tell a story… so the process was similar to my writing…but somehow…the images were more clear…less jargon…than when I write…which turns into a scrawl” – Juliette

The miracle question art activity was also introduced to students in a novel fashion, by handing students with a paper plate and asking them to select from a wide range of pre-cut images displayed on “the image buffet.” During the image selection process, students would frequently ask “how many images can I have?” with the researcher’s reply being “the buffet is all you can eat—but not literally.”

Such use of witticisms used by the therapist/researcher helped send the immediate message to students, from the outset of these encounters, that creative imagination, improvisation and humor were not only welcome but also highly encouraged. Highlighting the importance of such novel strategies, is the following comment made by a student named Cheryl:

• “It was so good to see all these pictures laid out on the buffet and crafts and stuff cause, um, it immediately tapped into that making side in my body and also the child-side” – Cheryl

In hindsight, using these novel strategies from the beginning helped to spark these creative students’ attention and interest needed for them to want to continue to engage in a process that required the exploration of problem stories. Students described how the tactile experience stimulated by the collage art making process was an important component of the SSAT encounter experience.

### Research Theme: SSAT as a “Tactile” Experience

All six students in this research project described SSAT as a “tactile experience.” More specifically, students described how the use of collage art materials along with the processes of art making such as gluing, cutting out and sticking down images, all provided a tactile means to stimulate implicitly held memories, thoughts and felt level impressions related to the problems they wished to process.

Gestalt psychologist Rudolf Arnheim previously argued that the embodiment of perceptions often exists in material form, in a process he called “thinking in medium” (Marshall, 2007). In a similar fashion, students in these SSAT encounters reported having glimpses of thoughts, feelings and emotions, stimulated by the art media, as captured in the following quotes:

• “I loved how sharp the black marker was…it suited the aggressiveness of the lines…it was a tactile experience…the stickiness of the black oil pastel was right for the tone of the anger that I was feeling” – Kate
• “The tactile nature of making the collage felt like I was playing…which I think was important for some reason…it’s like when you see a really serious issue framed in a humorous light…the art therapy was like that because I had permission to play with what was quite a serious issue…it freed it…loosened it up for me” – Joan

The tactile nature of the collage art making process can be likened to the “pre-intentional” phase of the phenomenological approach to art therapy (Betensky, 1995). During this pre-intentional phase, the art maker experiences glimpses of perceptions stimulated by the process of art making, but any felt level perceptions are yet to become consciously apparent to the art maker. Helping to bring these felt level glimpses into full view during these single session encounters, was the interactive process of inviting students to view and then describe the meanings of their artwork. Using Betensky’s (1995) question “what do you see?” during the intuiting phase of these encounters offered students a distanced means to begin to externalize problems.

### Research Theme: SSAT as “Getting the Problem Out”

Four out of six participants described SSAT as an experience as offering them a means for “getting the problem out.” Problem externalization is typically a verbal technique used in both narrative and single session therapies to help clients understand the nature of a problem, without seeing themselves as problematic (Combs and Freedman, 2012). Students described how the SSAT process afforded them a way to separate from problems in a bodily way, as revealed in the following student quotes:

• “Looking at my collage…it feels like I’ve taken something out of me, it’s like externalized and kind of exorcized” – Kate
• “I think the experience was actually like creating something and laying it out in front of me and having a removed perspective…and being like this isn’t just some issue that’s in here (*points to stomach*) or at my head and my heart or in my gut…. It felt like being able to physically remove the issue from my body…and then look at it not just in my head…and with someone with your eyes as well…and then dissect it…I think that process was really important to me” – Joan

Riley (1999) previously discovered how the process of viewing a problem from the distance of an artwork, can help concretize the understanding that the problem exists outside of the problem bearer. Many students in this study came with internalized views of their problem situations. Having the opportunity to separate from problems, using the safety of the artwork in these encounters, was important for helping students to see the broader sources of their distress. The important role of artistic metaphor also helped students to separate from seeing themselves as problems.

### Externalizing With Artistic Metaphors

According to Moon (2007), artistic metaphors are containers of multiple meaning and thus can provide opportunities for new ways of perceiving situations. Highlighting the role of artistic metaphor in aiding a process problem externalization, in this research study, is the following encounter story of a directing student named “Kate.”

### Encounter Story: Kate’s “Phoenix That Does Not Rise”

Kate entered the single session space wanting to problem solve what she called her “social awkwardness,” that she believed was the cause of her feeling as though she had not achieved “success” in her field of directing studies. Such an internalized view is encapsulated within Kate’s pre-session explanation of the problem:

• “I have always been a socially awkward person since childhood and now this has impacted my ability to make industry connections…to be a success in this field you need to be outgoing and bubbly…I want to reconcile this in some way” – Kate

**FIGURE 2:**
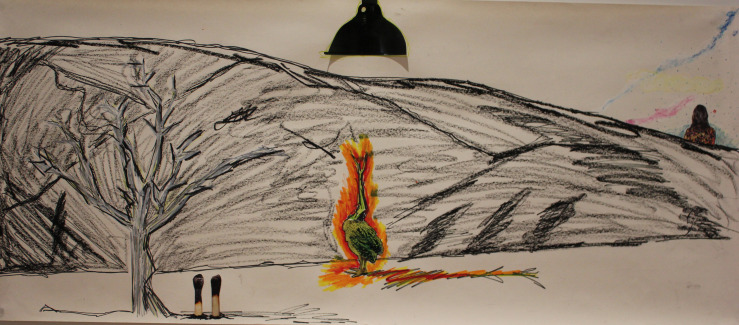
Participant artwork: “Phoenix that does not rise.”

During the collage art making process Kate used a combination of drawing with pastel and collage imagery, to create her own metaphorical image of a bird (Figure 2). Kate described the bird as a “*phoenix that doesn’t rise but is consumed and burnt up by fire*.” Asking Kate to describe her collage image led to her discovering feelings of anger. Viewing the image of her phoenix for a second time in the follow up interview, led Kate to making the following discoveries:

• “Looking at it now…I think there was an idea about being burnt out in it…and I suppose looking back…I can see in some ways…that I can choose to live in a way that might involve altering my expectations around what I can achieve…but that I don’t have to be there” (*points to the bird*) – Kate
• “At the end of the session…I was surprised that anger was there…for me anger is not my go to emotion…but knowing that anger is there…I suppose is like a positive because it allowed me to step back from the problem as being a problem with me and allows me to look at it as a problem with the industry” – Kate

Kate’s descriptions of her metaphorical image of the phoenix, highlights how perspective shifts can be stimulated when offering these students a means to look at problems through the safety of artistic metaphors. Providing Kate with a second viewing of her artwork during the post session follow up appeared to enable her to achieve a deeper clarity of perspective, regarding the broader outside sources of her anger. As Kate’s encounter story highlights, offering students in this study a safe means to “get the problem out” and then explore distressing content, through the distance of artistic metaphors, helped students to form a less internalized view of the self.

### Research Theme: SSAT as a “Seeing” Experience

Students described the SSAT experience as helping to open a space to “see” problems and possible solutions with a greater sense of clarity. According to Czamanski-Cohen and Weihs (2016) the ability to gain and shift perspectives in therapy can be useful in the promotion of mental health and wellbeing, as it can lead to the adoption of a less rigid and more flexible view of the self. Student descriptions of this “seeing” process stimulated during their SSAT encounter can be seen in the following quotes:

• “It wasn’t like how are you feeling or coping, it was more of an opening up of new ways of looking at the problem…it made me ask questions rather than form lockdown statements of facts”
• “Seeing that contrast in the collage helped me to see clearer…both the problem and the solution”
• “The process of seeing the image was like an opening up of new ideas”

### SSAT and Personal Sense of Agency

Four out of six students described how their encounter experience had helped them feel a personal sense of agency. Within these SSAT encounters, a deliberate effort was made by the therapist/researcher to follow the SST principle of engaging clients as “active agents” in control of their own encounter process. Therapeutic strategies such as asking students to describe what they would like to work on in the encounter, and then enabling students to locate and describe their own metaphors for problems, helped engage students as the “active experts” in charge of their own lives. The following quotes from the students highlight the sense of agency stimulated by the SSAT encounter:

• “After the session I felt like more empowered to be, like, yeah I could do this…the process of seeing what works has been helpful…and now I feel encouraged and supported in terms of like I know what to do and what works for me to manage stress” – Cheryl
• “Having that chance to make a collage…view it…and stand back on my own life is an act that felt quite empowering” – Joan

**FIGURE 3:**
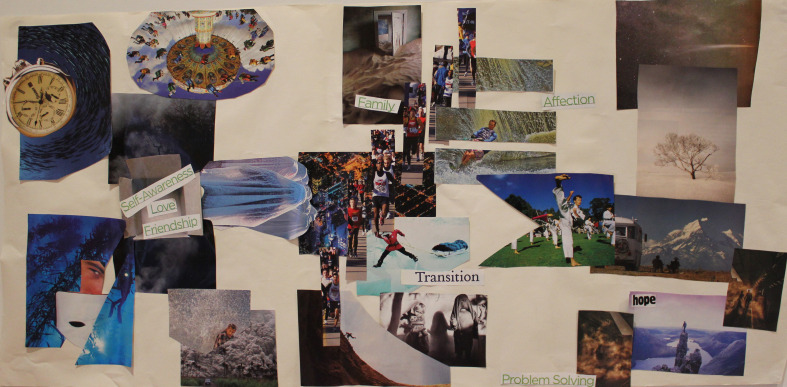
Participant artwork: “Holding out for love.”

According to Combs and Freedman (2012), offering opportunities inside the therapy space for clients to feel and see moments of mastery in relation to problems, can encourage people to make agentic changes outside of the therapy space. Within her follow up interview, a drama student named Juliette described how as a result of the SSAT experience, she had been encouraged her to use her own artistic metaphor (see Figure 3) as a way to communicate her distress with her boyfriend:

• “It was really empowering to use the art I made in the session… to then be able to tell my partner how the secrecy makes me feel “I’m in the crevice”…cause so often words are really inadequate to use and like we try and describe things and it ends up in this blurry vision world and so it was really nice to try to reach out to him through image” – Juliette

### Limitations

This was a small interpretive research study conducted with artistic university students with creative thinking abilities that could easily be harnessed via the use of the miracle question art activity. The combined single session art therapy approach used in this study is not a panacea to be used for all student mental health and wellbeing related concerns and could be inappropriate for some student populations. Given the small, exploratory and contextualized nature of this study, any discoveries from this research are not generalizable to other populations and settings. The purpose of this study was not to give therapists a recipe book on how to conduct single session art therapy; rather, the hope of this study is that it might spark a greater research interest in exploring brief and novel ways to reconfigure existing mental health and wellbeing services, in ways that hold the expressed preferences of students in mind.

Future research into the combination of art therapy within a single session framework could address the limitations of this study by using a quantitative design. Additionally, using a larger and more heterogeneous sample would ensure the generalizability of research findings.

## Data Availability Statement

The original contributions presented in the study are included in the article/supplementary material, further inquiries can be directed to the corresponding author.

## Ethics Statement

The studies involving human participants were reviewed and approved by the Human Research Ethics Office for Research Ethics and Integrity, University of Melbourne. The patients/participants provided their written informed consent to participate in this study. Written informed consent was obtained from the individual(s) for the publication of any potentially identifiable images or data included in this article.

## Author Contributions

The author confirms being the sole contributor of this work and has approved it for publication.

## Conflict of Interest

The author declares that the research was conducted in the absence of any commercial or financial relationships that could be construed as a potential conflict of interest.
